# Toll-Like Receptor 4 (TLR4)/Opioid Receptor Pathway Crosstalk and Impact on Opioid Analgesia, Immune Function, and Gastrointestinal Motility

**DOI:** 10.3389/fimmu.2020.01455

**Published:** 2020-07-08

**Authors:** Peng Zhang, Meirong Yang, Chunhua Chen, Liu Liu, Xinchuan Wei, Si Zeng

**Affiliations:** ^1^Department of Anesthesiology, Sichuan Academy of Medical Science and Sichuan Provincial People's Hospital, University of Electronic Science and Technology of China, Chengdu, China; ^2^Department of Anesthesiology, School of Medicine, Shanghai General Hospital, Shanghai Jiaotong University, Shanghai, China; ^3^Department of Anatomy and Embryology, School of Basic Medical Sciences, Peking University Health Science Center, Beijing, China

**Keywords:** TLR4—Toll-like receptor 4, opioid receptor, opioid tolerance and dependence, hyperalgesia, crosstalk

## Abstract

Toll-like receptor 4 (TLR4) recognizes exogenous pathogen-associated molecular patterns (PAMPs) and endogenous danger-associated molecular patterns (DAMPs) and initiates the innate immune response. Opioid receptors (μ, δ, and κ) activate inhibitory G-proteins and relieve pain. This review summarizes the following types of TLR4/opioid receptor pathway crosstalk: (a) Opioid receptor agonists non-stereoselectively activate the TLR4 signaling pathway in the central nervous system (CNS), in the absence of lipopolysaccharide (LPS). Opioids bind to TLR4, in a manner parallel to LPS, activating TLR4 signaling, which leads to nuclear factor kappa-light-chain-enhancer of activated B cells (NF-κB) expression and the production of the pro-inflammatory cytokines tumor necrosis factor (TNF)-α, interleukin (IL)-1β, and IL-6. (b) Opioid receptor agonists inhibit the LPS-induced TLR4 signaling pathway in peripheral immune cells. Opioids operate as pro-inflammatory cytokines, resulting in neuroinflammation in the CNS, but they mediate immunosuppressive effects in the peripheral immune system. It is apparent that TLR4/opioid receptor pathway crosstalk varies dependent on the cell type and activating stimulus. (c) Both the TLR4 and opioid receptor pathways activate the mitogen-activated protein kinase (MAPK) pathway. This crosstalk is located downstream of the TLR4 and opioid receptor signaling pathways. Furthermore, the classic opioid receptor can also produce pro-inflammatory effects in the CNS via MAPK signaling and induce neuroinflammation. (d) Opioid receptor agonists induce the production of high mobility group box 1 (HMGB1), an endogenous TLR4 agonist, supporting intercellular (neuron-to-glia or glia-to-neuron) interactions. This review also summarizes the potential effects of TLR4/opioid receptor pathway crosstalk on opioid analgesia, immune function, and gastrointestinal motility. Opioids non-stereoselectively activate the TLR4 pathway, and together with the subsequent release of pro-inflammatory cytokines such as IL-1 by glia, this TLR4 signaling initiates the central immune signaling response and modifies opioid pharmacodynamics. The DAMP HMGB1 is associated with the development of neuropathic pain. To explain morphine-induced persistent sensitization, a positive feedback loop has been proposed; this involves an initial morphine-induced amplified release of IL-1β and a subsequent exacerbated release of DAMPs, which increases the activation of TLR4 and the purinergic receptor P2X7R. Opioid receptor (μ, δ, and κ) agonists are involved in many aspects of immunosuppression. The intracellular TLR4/opioid receptor signaling pathway crosstalk induces the formation of the β-arrestin-2/TNF receptor-associated factor 6 (TRAF6) complex, which contributes to morphine-induced inhibition of LPS-induced TNF-α secretion in mast cells. A possible molecular mechanism is that the TLR4 pathway initially triggers the formation of the β-arrestin-2/TRAF6 complex, which is amplified by opioid receptor signaling, suggesting that β-arrestin-2 acts as a functional component of the TLR4 pathway.

## Introduction

Toll-like receptor 4 (TLR4) is a pattern recognition receptor belonging to the Toll-like receptor (TLR) family that contains an extracellular domain and an intracellular domain (1). TLR4 activates the innate immune response by recognizing pathogen-associated molecular patterns (PAMPs, including bacteria, viruses, fungi, and protozoa) or danger-associated molecular patterns (DAMPs, mainly endogenous signals for cell death and tissue damage) (2). Lipopolysaccharide (LPS), an outer surface component of Gram-negative bacteria, is an exogenous TLR4 agonist, while high mobility group box 1 (HMGB1) and heat shock proteins (HSPs) are endogenous TLR4 agonists (3). TLR4 signaling is roughly divided into two distinct pathways depending on the usage of the distinct adaptor molecules, myeloid differentiation primary response gene 88 (MyD88) and Toll-interleukin receptor-domain-containing adapter-inducing interferon-β (TRIF): the MyD88-dependent and TRIF-dependent (also known as MyD88-independent) signaling pathways (2, 3).

Opioid receptors belong to the seven-transmembrane G protein-coupled receptor (GPCR) superfamily, the members of which use G proteins for signal transduction (4). Opioid receptors are expressed throughout the nervous system and peripheral tissues and play critical roles in antinociception and pain management. There are three major subtypes of opioid receptor: mu (μ), delta (δ), and kappa (κ) opioid receptors (also called MOR, DOR, and KOR, respectively), among which MOR plays a predominant role in analgesia (5, 6). Opioid receptors are activated both by endogenous opioid peptides (dynorphin and enkephalin) and exogenous synthetic opioid drugs (morphine, fentanyl, and remifentanil) (7). After activation by agonists, multiple intracellular effects are initiated, including inhibition of adenylyl cyclases and cyclic adenosine monophosphate (cAMP), suppression of Ca^2+^ channels, stimulation of K^+^ channels, and activation of phospholipase C (PLC) and protein kinase C (PKC), which together inhibit presynaptic neurotransmitter release, induce postsynaptic hyperpolarization, and decrease neuronal excitability (5, 8).

It is apparent that the classic functions of the TLR4 and opioid receptor signaling pathways are remarkably distinct. Additionally, the stereoselectivity of opioid action at TLR4 and the opioid receptor is also different. To be specific, the opioid receptor is stereoselective, only binding to (–)-opioid isomers but not (+)-isomers, while TLR4 is non-stereoselective, binding to both opioid isomers (9, 10). However, Zhang *et al* recently reported that (+)-norbinaltorphimine [formed by coupling two pharmacophores derived from (+)-naltrexone] inhibited the LPS-induced TLR4 signaling pathway in microglia, astrocytes, and macrophages, whereas (–)-norbinaltorphimine did not, indicating that some xenobiotics show stereoselectivity for TLR4 (11). An early opioid-binding experiment by Goldstein et al. in 1971 found that there are saturable but non-classic non-stereoselective opioid-binding sites, which are much more abundant (~30-fold more abundant) than the classic stereoselective opioid-binding sites (12). This was the first evidence that opioids could non-stereoselectively bind to non-classic non-opioid receptors, although, for a long period of time, the findings of Goldstein et al. were considered to be experimental “noise” (13, 14).

In 1979, Wybran et al. reported that, based on active and total rosette tests, morphine inhibited human T lymphocytes, and this inhibition was completely reversed by the opioid receptor antagonist naloxone (15). This represents early evidence showing the immunosuppressive effects of opioids. Further evidence demonstrated that opioids suppress the immune system at various stages, starting from innate immune cells, encompassing antigen presentation, and ending with modulation of T lymphocyte activation and differentiation (16–18). The fact that MOR-knockout mice, unlike wildtype mice, did not show morphine-induced diminished natural killer (NK) cell activity indicated that MOR was implicated in immunosuppression (19). However, in 2005, Watkins et al. reported that spinal cord glia were activated and released neuroexcitatory substances in response to morphine, thereby inducing neuroinflammation and causing anti-analgesia effects, indicating a pro-inflammatory role for opioids in the central nervous system (CNS) (20). Further evidence collected during the last 10 years has confirmed that opioids also have pro-inflammatory effects in the CNS and induce the central immune response (21–23). Recognition of the involvement of TLR4 in opioid-induced central immune signaling arose from the early evidence that chronic intrathecal (+)-methadone and (+)-morphine (which have no affinity for the opioid receptor) induced glial activation and increased the expression of chemokines and cytokines in isolated dorsal spinal cords from rats (24).

In this review, we discuss the potential crosstalk between the TLR4 and opioid receptor signaling pathways and the implications of the crosstalk for opioid analgesia, immune function, and intestinal motility. Firstly, four aspects of TLR4/opioid crosstalk are discussed: (a) Opioid receptor agonists directly activate the TLR4 signaling pathway in the absence of LPS, indicating crosstalk within the cell membrane. (b) Opioid receptor agonists inhibit the LPS-induced TLR4 signaling pathway, indicating negative intracellular crosstalk. (c) Both the TLR4 and opioid receptor pathways activate the mitogen-activated protein kinase (MAPK) pathway, representing downstream crosstalk between the TLR4 and opioid receptor pathways. (d) Opioid receptor agonists induce the production of HMGB1, an endogenous TLR4 agonist, supporting intercellular (neuron-to-glia or glia-to-neuron) interactions. Secondly, we summarize and update current knowledge on opioid-induced central immune signaling and the effect of non-stereoselective TLR4 activation in the CNS on opioid analgesia; findings on the role of HMGB1 in maintaining morphine-induced persistent sensitization are also discussed. Thirdly, we summarize the peripheral immunosuppressive effect of opioids on innate immune cells, involving modulation of the immune system related to TLR4 signaling and LPS-activated immune cells. Fourthly, the differential involvement of TLRs (in intact animals vs. isolated colon segments) regarding morphine-induced inhibition of gastrointestinal transit are discussed.

## TLR4/Opioid Receptor Pathway Crosstalk

### Opioid Receptor Agonists Bind to TLR4 and Non-stereoselectively Activate TLR4

Many clinically relevant opioid receptor agonists, such as morphine, fentanyl, and oxycodone, bind to TLR4 by docking to the LPS-binding pocket of myeloid differentiation (MD)-2 (9, 21, 25, 26). Additionally, endogenous opioid peptides, for example, endomorphin (MOR), enkephalin (DOR), and dynorphin (KOR), and certain opioid metabolites are also TLR4 ligands (27–29). Morphine-3-glucuronide (M3G), an inactive metabolite of morphine, has little to no affinity for opioid receptors but enhances pain by activating the TLR4 signaling pathway (29). Naloxone and naltrexone are known as opioid receptor antagonists and are usually used to block the effects of opioids (30). Interestingly, acting as TLR4 antagonists, naloxone and naltrexone inhibit the opioid- or LPS-induced TLR4 signaling pathway (9, 25) and reverse TLR4-related neuropathic pain (31, 32).

Opioid receptor agonists bind to TLR4 and subsequently stimulate the TLR4 signaling pathway, which ultimately activates nuclear factor kappa-light-chain-enhancer of activated B cells (NF-κB) and releases pro-inflammatory cytokines (9, 21, 24, 25, 33). Wang et al. showed that, similar to LPS, morphine induced TLR4 dimerization and led to the formation of the (TLR4/MD-2)/(TLR4/MD-2) heterotetramer after docking with TLR4/MD-2 complexes (21). TLR4, MD-2, and MyD88 were found to be crucial for morphine-induced TLR4 pathway activation, as reduced production of NF-κB, interleukin (IL)-1β, and tumor necrosis factor (TNF)-α and inhibition of morphine-induced neuroinflammation were observed when TLR4, MD-2, or MyD88 was either knocked out or knocked down in *in vivo* and *in vitro* experiments (21). Moreover, the p38 and extracellular signal-regulated kinase (ERK) MAPK pathways are also involved in morphine-induced TLR4 pathway activation (21). Taken together, these findings show that opioids, like LPS, bind to TLR4 and activate the TLR4/MyD88-dependent pathway (including MAPK signaling cascades) (21). This extracellular interaction between opioids and TLR4 has mostly been observed in the CNS, including astrocytes, microglia, and endothelial cells, and it produces a pro-inflammatory effect and mediates neuroinflammation (13, 14, 23). It remains unclear whether this kind of crosstalk between opioids and TLR4 exists beyond the CNS.

As they do not express opioid receptors, HEK-Blue^TM^-hTLR4 cells (which are human embryonic kidney [HEK] 293 cells transfected with human TLR4 and related accessory proteins) are usually used to examine opioid effects targeted at TLR4 (25); TLR4 activity can be detected in these HEK-Blue^TM^ cells. Hutchinson et al. showed that, in the absence of LPS, nine opioids (morphine, methadone, M3G, etc.) at 10–100 μM non-stereoselectively activated the TLR4 signaling pathway, while naloxone and naltrexone did not (9). Moreover, the authors found that (–)-isomers (morphine and methadone) and (+)-isomers produced equivalent TLR4 activity, indicating that (+)-isomers and (–)-isomers have similar potency (9). Another two studies demonstrated that morphine at 3 and 10 μM, fentanyl at 0.3 μM, and M3G at 1–100 μM produced significant activation of the TLR4 pathway, while M6G (0.1–100 μM) did not (25, 33). Research has shown that LPS is the most potent agonist of TLR4 (9, 25, 33), while M3G is the second most potent. M3G is a consistent activator of TLR4 (M3G > 1 μM can activate the TLR4 pathway), while other opioid receptor agonists produce significant activation of the TLR4 pathway only at certain doses (9, 25, 33). Although these remaining opioid receptor agonists (including morphine, methadone, levorphanol, pethidine, buprenorphine, fentanyl, oxycodone, and dextrorphan) produced significant stimulation of TLR signaling (9), it is difficult to rank them in order, because of the limited data.

### Opioid Receptor Agonists *per se* Activate TLR4 but Inhibit LPS-Induced TLR4 Signaling Pathway Activation

In 2013, Stevens et al. reported that co-treatment of HEK-Blue^TM^ cells with morphine (3–100 μM) or fentanyl (1–100 μM) plus LPS (100 ng/ml) led to significant inhibition of TLR4 signaling activation in a non-competitive fashion, compared with LPS alone (25). Moreover, this inhibition was not blocked by an LPS antagonist (LPS-RS) or an opioid antagonist (naloxone or β-funaltrexamine [FNA]) (25). These findings are consistent with an *in vitro* experiment by Xie et al. (also using HEK-Blue^TM^ cells) and an *in vivo* experiment involving mice (33). The *in vitro* data showed that morphine and M3G (>1 μM) decreased LPS-induced TLR4 signaling activation (33). The *in vivo* data also supported this conclusion, as the plasma from morphine-treated mice inhibited LPS-induced TLR4 activation (33).

This phenomenon of opioids inhibiting LPS-induced TLR4 signaling activation is consistent with early studies (including on mast cells, human neutrophils, and human macrophages) (34–38) that showed that morphine and remifentanil inhibited LPS-induced production of TNF-α, IL-6, IL-8, IL-10, and IL-12 (34–38). Naloxone dose-dependently reversed the morphine-induced inhibition of LPS-induced TNF-α secretion in mice in a study by Bencsics et al. (36), while naltrexone did not prevent the decrease in LPS-induced IL-10 and IL-12 production in mice in a study by Limiroli et al. (35). In a study of human neutrophils, the p38 and ERK1/2 signaling pathways, but not c-jun N-terminal kinase (JNK) signaling, were implicated in remifentanil-induced inhibition of LPS-induced TLR4 signaling, and a KOR antagonist could reverse this inhibition (37). A further study by Madera-Salcedo et al. on bone marrow-derived mast cells proposed an underlying mechanism involving intercellular crosstalk between the TLR4 and opioid receptor pathways that induced the formation of an β-arrestin-2/TNF receptor-associated factor 6 (TRAF6) complex (34).

β-arrestins interact with certain TLR4 signaling molecules, such as IκB and TRAF6, and negatively regulate NF-κB activity (34, 39–41). In a study by Witherow et al., β-arrestin-1 and β-arrestin-2 bound to IκBα and subsequently attenuated NF-κB activity in transfected HeLa cells (39). Moreover, suppression of β-arrestin-1 expression using RNA interference led to a 3-fold increase in TNF-α-induced NF-κB activity (39). Gao et al. reported that activation of β_2_-adrenergic receptors (a type of GPCR) induced β-arrestin-2/IκBα formation, which inhibited the LPS/NF-κB signaling pathway and decreased IL-8 and TNF-α production in HEK293T cells (41). TRAF6 is a critical mediator of TLR/IL-1 signaling. β-arrestins can interact with TRAF6 and prevent TRAF6 autoubiquitination or oligomerization, which subsequently inhibits NF-κB and AP-1 activity, as shown in *in vitro* and *in vivo* experiments (40).

In the study by Madera-Salcedo et al., morphine treatment of mast cells prevented the production of the LPS-induced pro-inflammatory cytokine TNF-α and the activation of the TLR4 signaling molecules ERK1/2 and IKK (both of which belong to the MyD88-dependent pathway) (34). There were also morphine-induced decreases in TRAF6 ubiquitination and TRAF-activated kinase 1 (TAK1) phosphorylation (34). Given that β-arrestin operates as a negative regulator of the TLR4 pathway, unsurprisingly, morphine and LPS co-treatment induced the formation of the β-arrestin-2/TRAF6 complex in the mast cells, which subsequently inhibited the TLR4 signaling pathway. Only the combination of MOR and DOR antagonists could reverse the morphine-induced inhibition of LPS-induced secretion of TNF-α in mast cells, indicating that MOR/DOR heterodimers may be implicated in this antagonism (34).

Unfortunately, there is currently no evidence regarding whether this intracellular negative crosstalk (opioid-induced inhibition of LPS-induced TLR4 pathway activation) exists in other cell types. Although TLR4 initiates the innate immune response, the extent to which this negative TLR4/opioid crosstalk participates in opioid-induced immunosuppression is also unclear. It is apparent that the phenotypes related to TLR4/opioid receptor pathway crosstalk are complicated and varied dependent on the cell type or cellular microenvironment. In the CNS, opioids non-stereoselectively activate TLR4 and operate as pro-inflammatory cytokines, thereby resulting in neuroinflammation (21–23). In contrast, in mast cells or other peripheral immune cells, opioids inhibit LPS-induced TLR4 pathway activation (34–38) and mediate peripheral immunosuppressive effects (18). We infer that the cell function and stimuli likely determines the phenotype that TLR4/opioid crosstalk will initiate. In the future, more studies are needed to investigate the precise mechanisms.

### Both the TLR4 and Opioid Receptor Pathways Activate the MAPK Pathway

The MAPK pathway includes a range of proteins such as p38, ERK, and JNK, which are involved in many facets of cellular regulation, from gene expression to cell death (42). In the TLR4/MyD88-dependent signaling pathway, MyD88 activates TRAF6 and TAK1. Next, TAK1 activates p38, ERK, and JNK, which subsequently activate activator protein 1 (AP-1) and produce pro-inflammatory cytokines, thereby mediating the inflammatory response (2, 43–45).

The opioid receptor is primarily controlled by interactions with two proteins: G proteins and β-arrestins, which initiate G protein signaling and β-arrestin signaling, respectively (46–48). Evidence shows that both the G protein and β-arrestin pathways can activate MAPK (8, 49, 50). Acute ultra-low-dose morphine upregulated spinal phosphorylation of JNK1, JNK2, and c-Jun, and activated spinal astrocytes, which were inhibited by naloxone, MOR silencing, and a JNK inhibitor (51). The spinal JNK activated by PKC also contributed to morphine thermal hyperalgesia (51). Xie et al. showed that morphine-induced apoptosis of microglia was mediated by the GSK-3β and p38 MAPK pathways in an opioid receptor-dependent manner (52). In hippocampal neural progenitor cell lineages, ERK was also activated by morphine and fentanyl via the PKC-dependent and β-arrestin-dependent pathways, respectively (50).

The TLR4-induced MAPK pathway can initiate immune and inflammatory responses, defending against harmful stimuli (2, 43–45), while the opioid receptor-induced pathway is more complicated. Merighi et al. showed that, in activated mouse microglia, morphine acted as a pro-inflammatory mediator and induced the production of nitric oxide (NO), TNF-a, IL-1β, and IL-6 via the PKC-Akt-ERK1/2 signaling pathway in a MOR-dependent manner (53). Subsequently, the same group found that, in activated microglia treated with low-dose morphine, NF-κB was a downstream component of the PKC-Akt-ERK1/2 signaling pathway (54). As discussed above, spinal astrocytes were activated via the MOR-PKC-JNK signaling pathway and were involved in the contribution of morphine to thermal hyperalgesia (51). The p38 MAPK pathway has also been linked to microglial activation and it contributed to postoperative thermal hyperalgesia and mechanical allodynia in rats (55) and morphine tolerance (56). Therefore, in glia, the intracellular TLR4/opioid receptor pathway crosstalk involves the MAPK pathway, which mediates the pro-inflammatory response and modifies the opioid analgesia effect (13, 14).

### Opioids Induce HMGB1 Production

HMGB1 is a DNA-binding protein and is abundant in the cell nucleus (57). HMGB1 is an endogenous agonist of TLR4. During activation or cell death, HMGB1 translocates from the nucleus to the cytoplasm or extracellular space (57, 58). Extracellular HMGB1 binds to and stimulates a variety of receptors, including the receptor for advanced glycosylation end products (RAGE), TLR2, TLR4, TLR5, CD24, and other receptors (58–63). The HMGB1-RAGE signaling pathway was the first demonstrated pathway implicated in cell growth, migration, differentiation, and up-regulation of cell-surface receptors in endothelial and somatic cells (58). In addition, HMGB1-TLR4 signaling initiates the innate immune response, which activates NF-κB and produces cytokines such as TNF-α, IL-1β, and IL-6 in macrophages, monocytes, and glial cells (62, 63).

HMGB1 is passively released from necrotic or damaged cells or actively secreted by stimulated immune cells (57, 59). In macrophages and monocytes, HMGB1 was found to be released after stimulation with LPS, TNF-α, or IL-1β (64, 65). Notably, a study of chronic intrathecal injection of morphine showed that the expression of HMGB1, TLR4, and RAGE in the rat spinal dorsal horn increased (66), while another study of a neuropathic pain model showed that subcutaneous administration of morphine increased HMGB1 expression even at 5 weeks after morphine was ceased (67). In these two studies of morphine, only extracellular HMGB1, acting as a pro-inflammatory mediator, HMGB1 in the media also increased (66, 67). Taken together, these findings indicate that morphine increases the expression and release of HMGB (66, 67). Studies investigating the underlying mechanism demonstrated that TLR4, P2X7R, caspase-1 antagonists, and TLR4 siRNA inhibited the increased levels of HMGB1, while opioid receptor antagonists did not (66). Therefore, TLR4 may partially mediate morphine-induced HMGB1 production (66, 67).

## TLR4/Opioid Receptor Pathway Crosstalk, Central Immune Signaling, and Opioid Analgesia

Opioids are used to treat severe pain, but they can also cause anti-analgesic effects, resulting in tolerance, hyperalgesia, or allodynia (68). Previous reviews highlighted that opioid-induced central immune signaling contributed to decreased opioid analgesic efficacy (13, 14, 23). In this section, we summarize the main opinions on opioid-induced central immune responses (13, 14, 23): (a) Non-neuronal immunocompetent cells (mainly astrocytes and microglia) in the CNS play a critical role in opioid-induced central immune signaling, modifying opioid pharmacodynamics by mediating pro-inflammatory reactivity. (b) The opioid-induced central immune signaling events include the release of a variety of immune molecules such as IL-1, TNF-α, IL-6, CCL2, CX3CL1, ATP, and NO, disruption of glutamate homeostasis, and increased neuronal excitability, which subsequently attenuate opioid analgesic efficacy. (c) Many intracellular signaling pathways are involved in opioid-induced neuroinflammation; the most prominently reported ones are the TLR4, MAPK, inositol trisphosphate (IP3)/Akt, and ceramide/sphingosine signaling pathways. Both classic opioid receptors and non-opioid receptors participate in this opioid-induced cellular adaptation. (d) *in vivo, in vitro*, and *in silico* approaches have demonstrated that opioids bind to TLR4 and non-stereoselectively activate the TLR4 signaling pathway. This non-stereoselective opioid activation of TLR4 triggers glial reactivity, which induces the release of neuroexcitatory immune mediators that play key roles in neuroinflammation.

The non-stereoselective response and opioid-induced hyperalgesia still observed in triple opioid receptor (MOR, DOR, and KOR)-knockout mice suggests that non-stereoselective non-classic opioid actions are implicated in opioid analgesia in these studies (10, 69, 70). At least some of these actions have been attributed to TLR4 (9, 13, 14, 21, 23). A diversity of clinically relevant opioids can bind to the TLR4/MD2 heterodimer, induce TLR4 oligomerization, and trigger a pro-inflammatory response, thereby resulting in neuroinflammation (21). Additionally, acute blockade (71, 72), genetic mutation (73), and knockout (74) of TLR4 each resulted in a significant potentiation of the magnitude and duration of opioid analgesia, compared with the observations in control animals.

However, evidence also shows that opioid tolerance and hyperalgesia were still retained in TLR4-mutant and -knockout mice (75–77). Nevertheless, findings from these mice, with regard to the influence of TLR4 on nociception, must be interpreted with caution and require further investigation. This is because of two findings: (a) some TLR4 agonists have been found to signal around TLR4 mutation (78) and (b) TLR4 is by no means the only receptor that mediates glial activation, and compensatory pathways may be activated in the absence of TLR4 (79). Hutchinson et al. believe that opioid-induced TLR4 signaling initially triggers opioid-induced central immune signaling (13); this does not mean that all opioid-induced neuronal activity depends on TLR4, but rather that this activity is complemented and facilitated by the TLR4 pathway (13).

HMGB1 is considered to be a pro-inflammatory cytokine and it is significantly expressed in rats with neuropathic pain caused by partial sciatic nerve ligation (80). Anti-HMGB1 monoclonal antibody significantly attenuated hind paw tactile hypersensitivity in these rats (80). Aside from neuropathic pain, increased HMGB1 has also been linked to other types of chronic pain including diabetic, arthritic, and cancer-induced pain (81–83). In a diabetic pain model, which involved the development of persistent mechanical allodynia, HMGB1 was significantly increased and anti-HMGB1 antibody inhibited mechanical allodynia (81). There is also other evidence demonstrating the critical role of HMGB1 in abnormal pain processing. Intrathecal, intraplantar, and perineural injection of HMGB1 produced mechanical hypersensitivity (62, 84, 85). HMGB1 is a multifunctional protein that interacts with a variety of receptors. Tolerance, hyperalgesia, and allodynia have been shown to involve HMGB1 activating the RAGE, TLR4, and TLR5 signaling pathways (60, 61, 84, 86).

In a mouse model of neuropathic pain, morphine has recently been reported to prolong the duration of mechanical allodynia for months after morphine treatment was ceased (87). The authors demonstrated that the prolonged neuropathic pain arose from activated spinal microglia, release of IL-1, and the NOD-like receptor protein 3 (NLRP3) inflammasome, a protein complex that activates IL-1β via caspase-1 (87). The amplification of spinal microglial activation may be explained by the “two-hit hypothesis,” with nerve injury being the first “hit” and morphine treatment the second (87). However, the question is how spinal NLRP3 inflammasome signaling is continuously activated long after morphine treatment is stopped. In another study, Grace et al. concluded that morphine treatment leads to persistent release of DAMPs (including HMGB1 and biglycan) via TLR4, the purinergic receptor P2X7R, and caspase-1, and these DAMPs are involved in continuous NLRP3 inflammasome activation (67). There is a positive feedback loop that maintains the NLRP3 inflammasome activation, which begins with morphine-induced amplified release of IL-1β and ends with disruption of glutamate homeostasis and exacerbated release of DAMPs that increase the activation of TLR4 and P2X7R to maintain persistent NLRP3 inflammasome activation (67, 87). Opioid non-stereoselective activation of TLR4, together with the release of DAMPs that increase the activation of TLR4 and P2X7R signaling, may provide a critical initiating trigger for continuous NLRP3 inflammasome activation (13, 67).

## TLR4/Opioid Receptor Pathway Crosstalk and the Immune Response

Opioid administration has been shown to inhibit the innate and adaptive immune systems at different stages, increasing the risk of opportunistic infection (16, 17, 88). Opioid-induced immunosuppression can be mediated directly via inhibition of immune cells and/or through indirect interaction with the hypothalamic–pituitary–adrenal (HPA) axis and the sympathetic nervous system (16, 17, 88). However, the precise cellular mechanisms underlying the immunosuppressive effects of opioids are largely unknown.

In 1998, Gavériaux-Ruff et al. observed that in wildtype mice, but not MOR-deficient mice, morphine treatment led to compromised immune responses (lymphoid organ atrophy, a diminished ratio of CD4^+^/CD8^+^ cells in the thymus, and reduced natural killer activity) (19). Research using pharmacological antagonists and MOR-knockout mice confirmed that MOR participated in opioid-induced immunosuppression (18). TLR4 and opioid receptors are co-expressed in immune cells, and TLR4 has a key role in the innate immune response, so TLR4 may also be linked to opioid-induced immunosuppression. In this section, we summarize the opioid modulation of the immune system involving the TLR4 signaling pathway. As LPS acts solely through TLR4, research on LPS-activated immune cells is also included.

As shown in Table 1, MOR activation inhibited LPS-induced NF-κB DNA-binding in a NO-dependent mechanism in human neutrophils and monocytes (99). Additionally, MOR stimulation suppressed the LPS-induced p38 and ERK1/2 pathways in neutrophils (37). MOR agonists also inhibited the LPS-induced production of NO (13, 92) and prostaglandin E2 (PGE2) (94) and secretion of the pro-inflammatory cytokines IFN-α (94), TNF-α (92), and IL-8 (37, 98). Moreover, MOR agonists reduced LPS-induced macrophage viability (92), inhibited the capacity of macrophages and monocytes to respond to LPS (89, 94), and suppressed NK cell cytotoxicity (100) in both *in vitro* and *in vivo* studies. There have been some controversial studies on the MOR-induced expression of TLR4 mRNA and protein in macrophages (89, 90) and IL-6 production in neutrophils and NK cells (37, 100), as some studies indicated increases and other studies indicated decreases. Morphine, in the presence of LPS, has been shown to prevent macrophage and neutrophil recruitment to wound sites, which decreased wound closure and wound integrity and increased bacterial sepsis (93). In a study by Wan et al., although morphine facilitated macrophage autophagy initiation through the TLR4/p38 pathway, it also inhibited autophagolysosomal fusion, which decreased the bacterial clearance and increased the bacterial load (91).

**Table 1:** Effect of TLR4/opioid receptor pathway crosstalk in peripheral immune cells.

**Opioid receptor**	**Cell type**	***Vivo*/*vitro***	**Pathway**	**Immunomodulatory effects**	**Inhibitor**
MOR	Macrophages	BMDM, RAW 264.7, J774.1 cells; C57, TLR4/MOR knockout mice	Increase or decrease TLR4 mRNA and protein expression (89, 90). Potentiate autophagy initiation through TLR4/p38 pathway, but inhibit autophagosomal maturation though MOR pathway (91). Suppress LPS-activated NO and TNF-α production (92)	Compromise the capacity of macrophages to respond to LPS (89). Reduce the cell viability (92) and bacterial clearance (91). Increased bacterial load (91) and bacterial sepsis (93). Prevent macrophage recruitment to the wound site and decrease the wound closure and wound integrity (93)	Naltrexone (89), PTX (89)
	Monocytes	THP-1 and other cells	Suppress LPS-induced IFN-α and PGE2 production (94). Inhibit LPS-stimulated IL-10, IL-12 (95), and arachidonic acid, PGE2, ROI, and NO_2_ production (96). Potentiate LPS-stimulated NF-κB DNA binding (95)	Decrease antiviral defense and inhibit their response to activating stimuli (94). Inhibit LPS-stimulated monocyte activation (95) and instauration of a hyporesponsive phenotype on DC development (96)	
	Mast cells	BMMCs cells, C57; MS deficient/reconstituted mice	Inhibit LPS-induced TNF-α (34, 38, 97) but not CCL2 release (38)	Resident mast cells mediate selective morphine immunosuppression (38)	
	Neutrophils	*vitro*	Inhibit LPS-induced p38, ERK1/2 pathway activation (37) and decrease TNF-α, IL-6 (37), and IL-8 production (37, 98). Inhibit LPS-induced NF-κB binding in a NO-dependent mechanism (99)	Reduce neutrophils recruitment to the wound site and decrease the wound closure and wound integrity and increase bacterial sepsis (93)	KOR antagonist (37), naloxone (98)
	NK cells	*vitro*	Increase IL-6 (naloxone) and granzymes A and B (TAK-242) production (100)	Decrease NK cell ability to induce apoptosis in K562 cells and suppress NK cell cytotoxic activity (100)	Naloxone (100), TAK-242 (100)
DOR	Macrophages	RAW 264.7 cells; sepsis rat model	Increase LPS-induced TNF-α and NO production (101). Suppress LPS-induced release of HMGB (102). DOR2: inhibit p38 MAPK activation and expression of TNF-α and MIP-2 (103)	Potentiate LPS-stimulated macrophage functions (101). Suppress LPS-induced cell death and protect rats from sepsis (102)	
KOR	Macrophages	J774 and other cells	Inhibit LPS-stimulated nitrite (104, 105), TNF-α (104, 105), IL-10 (104) and iNOS (104), IL-1 (105) and IL-6 production (105). Decrease NO release (106)	Moderate anti-inflammatory effects (104). Inhibit the cytotoxicity of macrophages (106)	Naloxone (104), naloxone (partially) (105), norBNI (104, 105)
	Monocytes	P388D1 and THP1 cells	Suppress LPS-stimulated IL-6 production (107). Inhibit LPS-induced NF-κB/p65 nuclear translocation and IL-1β, TNF-α release (108)	Anti-inflammatory effect (108)	nor-BNI (107), ML-190 (108)
	Neutrophils	Ischemia–reperfusion injured rat heart model	Attenuate the expressions of TLR4, NF-κB and TNF-α (109)	Inhibit neutrophil accumulation (109). Cardioprotective and anti-inflammatory effects (109)	nor-BNI (109)

*MOR, μ opioid receptor; DOR, δ opioid receptor; KOR, κ opioid receptor; BMDM, bone marrow-derived macrophages; RAW264.7 cells, mouse leukemic monocyte macrophage cell line; BMMC, bone marrow–derived mast cells; K562 cells, a chronic myelogenous leukemia-derived; P388D1 cells, a mouse monocyte-like cell line; THP-1, human monocytic cell line; NK, natural killer cells, MOR agonists, morphine, fentanyl, remifentanil, DAMGO, and endomorphin 1/2; DOR agonists, DADLE, SNC 80, and Deltorphin-dvariant; KOR agonists, Salvinorin A, U50488H, and dynorphin 1–17; norBNI, nor-binaltorphimine (a KOR-selective antagonist); ML-190, a selective KOP receptor antagonist; TAK-242 (TLR4 signaling antagonist)*.

In 2009, Li et al. showed that morphine-induced apoptosis was mediated via the TLR2 signaling pathway in HEK293 cells (110). Moreover, inhibition of MyD88 or overexpression of β-arrestin-2 attenuated morphine-induced apoptosis in TLR2-overexpressing HEK293 cells (110). The findings demonstrated that β-arrestin-2 negatively regulated morphine-induced TLR2-mediated apoptosis (110). However, the possible molecular mechanism was not explored in the study by Li *et al*. As previously mentioned, Madera-Salcedo et al. found that morphine-induced inhibition of LPS-induced TNF-α production was associated with the formation of β-arrestin-2/TRAF6 complex in bone marrow-derived mast cells (34). As a negative regulator of the TLR pathway (39–41), the findings indicated that β-arrestins also contribute to opioid-induced immunosuppression (34, 110). In the study by Madera-Salcedo et al., LPS stimulation led to the formation of the β-arrestin-2/TRAF6 complex, which was amplified by co-treatment with morphine (34). Furthermore, to some extent, this conclusion is consistent with a study published in 2006 showing that activation of the TLR/IL receptor increased β-arrestin-2/TRAF6 formation, but stimulation of the β_2_-adrenergic receptor (a type of GPCR) did not, indicating that β-arrestins act as the intrinsic signaling molecules of the TLR/IL pathway (40). Therefore, β-arrestins, operating as a functional component of the TLR4 pathway, initiate the formation of the β-arrestin-2/TRAF6 complex; subsequently, the formation is amplified by opioid receptor signaling, which is thus implicated in the LPS-induced TLR4 signaling pathway.

On the other hand, NF-κB essential modulator (NEMO), acting as a regulatory subunit of the NF-κB complex, is also another important target site for regulating NF-κB activity. Tripartite interaction motif 29 (TRIM29) is a key negative regulator of NF-κB activity, which functions via direct ubiquitination and proteolytic degradation of NEMO, which negatively regulates the production of type I interferons as well as pro-inflammatory cytokines in alveolar macrophages after infection (111). TRIM29 has been reported to inhibit the activation of the innate immune system (111, 112). Further studies are required to explore whether TRIM29 is involved in the opioid-induced inhibition of the LPS-induced TLR4 signaling pathway.

## TLR4/Opioid Receptor Pathway Crosstalk and Intestinal Function

Constipation is the most common gastrointestinal side effect of opioids, occurring in 40–95% of patients (113). For 30 years, the opioid receptor was considered to exclusively mediate the morphine-induced inhibition of gastrointestinal transit. The supporting evidence was that MOR antagonist (naloxone) and MOR-knockout technology could abolish morphine-induced inhibition of gastrointestinal transit (114, 115). However, TLR4 is widely expressed within the gastrointestinal tract and is associated with irritable bowel syndrome (116) and inflammatory bowel disease (117, 118), which are characterized by gut dysmotility. In 2015, using TAK-242 (a selective TLR4 antagonist), Farzi et al. demonstrated that TLR4 was also involved in morphine-induced depression of peristalsis in isolated guinea pig colons *in vitro* and it was also involved in inhibition of colorectal propulsion in mice *in vivo* (119).

However, the effects of TLR4 regarding opioid-induced inhibition of gastrointestinal transit are complicated. Farzi et al. found that TLR4 antagonism using TAK-242 failed to prevent morphine-induced inhibition of peristalsis in gastrointestinal regions besides the colorectum *in vivo* and *in vitro* (119). These findings indicated that TLR4/opioid receptor pathway crosstalk varies along the gastrointestinal tract. To some extent, the study by Farzi et al. was consistent with a study by Beckett et al. Using knockout technology, Beckett et al. showed that TLRs (TLR2 and TLR4) and the adaptor protein MyD88 participated in morphine-induced slowed movement of ingested content in mice, while *in vitro* results based on isolated colons did not support the involvement of TLRs (120); they hypothesized that TLR signaling pathways extrinsic to the colon may explain the differential involvement of TLRs (in intact animals vs. isolated colon segments) regarding the morphine-induced inhibition of the transit of ingested content (120). However, this hypothesis seems inconsistent with the well-accepted paradigm that the peripheral MOR expressed on intrinsic enteric neurons predominantly explains the phenomenon of opioid-induced constipation (121, 122), although there is still evidence supporting a central mechanism (123). Further studies are required to explore whether a peripheral mechanism vs. a central mechanism, or a combination of both, mediate the differential effects of morphine without TLR receptor signaling.

It is not an easy task to elucidate the mechanism underlying the inhibition of gastrointestinal transit. In the CNS, opioids have been demonstrated to directly bind to TLR4 and non-stereoselectively activate the TLR4 signaling pathway, which subsequently activates glial cells and initiates the immune response (13). Unfortunately, to date, not enough evidence has confirmed that non-stereoselective activation of TLR4 by opioids is also involved in gastrointestinal transit (119). Likewise, it is not wise to reject this possibility (119). Another explanation is that TLRs might be important functional components of the opioid receptor signaling pathway, and the two signaling events could interact with each other without direct binding of opioids to TLRs in the digestive system (119, 120, 124). The supporting evidence is that the opioid receptor pathway has been shown to synergize with the TLR4 pathway to impair the intestinal barrier function and increase bacterial translocation (124). In contrast, blocking the TLR pathway (either pharmacologically or using a genetic approach) elicits upon the actions of opioid agonists (119, 120). Further studies are needed to examine these hypotheses.

## Author Contributions

PZ and SZ designed and wrote the manuscript. MY and CC revised the manuscript. LL and XW generated the table. All authors contributed to the article and approved the submitted version.

## Conflict of Interest

The authors declare that the research was conducted in the absence of any commercial or financial relationships that could be construed as a potential conflict of interest.
